# Utility of Saline-Induced Resting Full-Cycle Ratio Compared with Resting Full-Cycle Ratio and Fractional Flow Reserve

**DOI:** 10.1155/2020/5787439

**Published:** 2020-04-06

**Authors:** Takao Sato, Sonoka Goto, Yusuke Ohta, Yuji Taya, Sho Yuasa, Minoru Takahashi, Masaaki Okabe, Yoshifusa Aizawa

**Affiliations:** Cardiology, Tachikawa General Hospital, Nagaoka, Japan

## Abstract

**Background:**

The saline-induced distal coronary pressure/aortic pressure ratio predicted fractional flow reserve (FFR). The resting full-cycle ratio (RFR) represents the maximal relative pressure difference in a cardiac cycle. Therefore, the present study aimed to compare the results of saline-induced RFR (sRFR) with FFR.

**Methods:**

Seventy consecutive lesions with only moderate stenosis were included. The FFR, RFR, and sRFR values were compared. The sRFR was assessed using an intracoronary bolus infusion of saline (2  mL/s) for five heartbeats. The FFR was obtained after an intravenous injection of papaverine.

**Results:**

Overall, the FFR, sRFR, and RFR values were 0.78 ± 0.12, 0.79 ± 0.13, and 0.83 ± 0.14, respectively. With regard to anatomical morphology were 40, 18, and 12 cases of focal, diffuse, and tandem lesion. There was a significant correlation between the sRFR and FFR (*R* = 0.96, *p* < 0.01). There were also significant correlations between the sRFR and FFR in the left coronary and right coronary artery (*R* = 0.95, *p* < 0.01 and *R* = 0.98, *p* < 0.01). Furthermore, significant correlations between sRFR and FFR were observed in not only focal but also in nonfocal lesion including tandem and diffuse lesions (*R* = 0.93, *p* < 0.01 and *R* = 0.97, *p* < 0.01). A close agreement on FFR and sRFR was shown using the Bland–Altman analysis (95% CI of agreement: −0.08–0.07). In the receiver operating characteristic curve analysis, the cutoff value of sRFR to predict an FFR of 0.80 was 0.81 (area under curve, 0.97; sensitivity 90.6%; and specificity 98.2%).

**Conclusion:**

The sRFR can accurately and safely predict the FFR and might be effective for diagnosing ischemia.

## 1. Introduction

The pressure-derived fractional flow reserve (FFR) index is a standard method for evaluating the functional significance of epicardial coronary artery stenosis, and clinical outcomes of FFR-guided percutaneous coronary intervention (PCI) are better than those of angiography-guided PCI or medical treatment [1–3]. To achieve maximal hyperemia for FFR assessment, an intravenous administration or intracoronary high-dose bolus of adenosine has been employed [4–6]. However, completion of one FFR measurement using an intravenous infusion of adenosine requires 4 to 5 minutes, and an intracoronary bolus of adenosine has been reported to have some potential drawbacks [4–6]. In addition, a previous study also reported the incidence of complications caused by papaverine, such as ventricular fibrillation and QT prolongation [7]. Although contrast-based FFR provided effective diagnostic performance for predicting FFR [8], in actual clinical practice, the use of an additional contrast is often not preferred in cases with renal insufficiency. Recently, the saline-induced distal coronary pressure (Pd)/aortic pressure (Pa) ratio has been used to predict the functional significance of coronary stenosis assessed using FFR [9].

The resting full-cycle ratio (RFR) is a novel hyperemia-free resting measurement parameter, which measures pressure at the point of the absolute lowest resting Pd to Pa ratio during the cardiac cycle. The RFR represents the maximal relative pressure difference in the cardiac cycle completely independent of electrocardiography and irrespective of systole or diastole, thus being an unbiased physiological assessment of coronary artery stenosis. However, it remains unclear whether saline-induced RFR (sRFR) can predict FFR. Therefore, the present study aimed to compare the results of saline-induced RFR with FFR.

## 2. Methods

### 2.1. Study Population

Seventy consecutive cardiac lesions with more than moderate stenosis (exceeding 30%) based on visual estimation by coronary angiography were included in this study. The FFR, RFR, and sRFR were measured to identify functionally significant stenosis. Exclusion criteria were as follows: (1) left main coronary artery disease, (2) acute myocardial infarction within the preceding 2 weeks, (3) severe valvular heart disease, (4) decompensated congestive heart failure, (5) hemodynamic instability, (5) acute coronary syndrome, and (6) atrial fibrillation (AF).

### 2.2. Coronary Angiography

**Figure 1:**
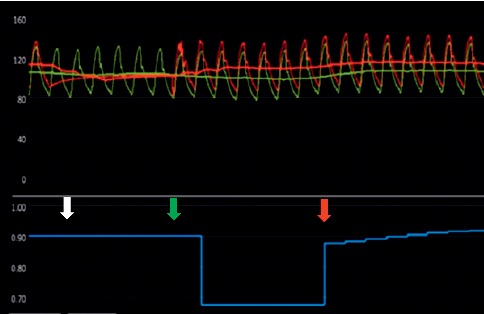
A representative data. Saline-induced resting full-cycle ratio (sRFR) record from a 62-year-old man with stable angina. The red-arrow shows sRFR value in the present case. The white arrow shows the starting point of saline injection and the green arrow shows the ending point of saline injection.

Coronary angiography was performed using a 5-French (Fr) diagnostic or 6-Fr guiding catheter. We intravenously administered 100 IU/kg of heparin before coronary angiography was performed. We used a nonionic contrast medium (Iopamiron®; Bracco, Milan, Italy). Two experienced cardiologists visually assessed the severity of coronary stenosis. Quantitative coronary angiography was performed in optimal projections with validated software (CAAS II, Pie Medical Imaging, Maastricht, Netherlands). In addition, lesions were classified into 3 types such as the following: focal, tandem, or diffuse. Angiographic focal lesion was defined as stenosis measuring <20  mm long, angiographic diffuse lesion as stenosis measuring ≥20  mm long, and tandem lesion as 2 or more stenoses separated by an angiographically normal appearing segment of ≥20  mm in one epicardial coronary artery [10–12] (Figure 1).

### 2.3. Pressure Measurements

After calibration and equalization of a 0.014-inch pressure guidewire (Certus™ and Aeris™; St. Jude Medical/Abbott, St. Paul, MN, USA), the wire was advanced into the site distal to the stenosis. The study procedure was as follows: First, the RFR was automatically measured at resting status twice. The mean of the two values was adopted as the RFR value. Second, after the RFR measurement, the sRFR was assessed by an intracoronary bolus of saline at room temperature at 2  mL/second for 5 heartbeats through the catheter using a power injector system (ACIST®; ACIST Medical Systems, Eden Prairie, MN, USA) twice. The sRFR was recorded in live mode. The sRFR value was defined at the inflection point between the rapid increase and plateau of the RFR value, which represented the RFR value in one beat. Third, FFR was finally assessed during peak hyperemia by using an intracoronary infusion of papaverine. Papaverine was administered into the coronary artery: 12  mg into the left coronary artery (LCA) and 8  mg into the right coronary artery (RCA), which induced maximal dilatation within 15  seconds. Thereafter, the FFR value was assessed during peak hyperemia.

### 2.4. Ethical Statement

Our study was approved by our institutional ethics review board, and all participants provided written informed consent about this protocol for the use of their data in our prospective analysis.

### 2.5. Statistical Analyses

All statistical analyses were performed using SPSS version 22 (IBM Japan, Tokyo, Japan). Continuous data with a nonnormal distribution are presented as means ± standard deviations, and categorical data are presented as counts and percentages. The correlations between 2 factors among sRFR, RFR, Pd/Pa, and FFR values were evaluated using the Spearman rank correlation coefficient.

In addition, the receiver operating characteristic (ROC) curve analysis was used to identify the sRFR cutoff value for predicting an FFR value ≤0.80. Furthermore, we analyzed the agreement between the sRFR and FFR using Bland–Altman plots and 95% limits of agreement. A two-sided *p* value <0.05 was considered statistically significant in all analyses (Table 1).

## 3. Results

### 3.1. Baseline Characteristics

Subjects' mean age was 72 ± 9 years. 76% were men. In addition, with regard to anatomical morphology were 40, 18, and 12 cases of focal, diffuse, and tandem lesion, respectively. Subjects with acute coronary syndrome were not included. The numbers of lesions in the left anterior descending (LAD), left circumflex (LCX), and RCA were 37 (53%), 15 (21%), and 18 (26%), respectively. Overall, the minimum coronary lumen diameter and percent diameter stenosis on quantitative coronary angiography were 1.16 ± 0.53  mm and 55.0 ± 16.4%, respectively.

### 3.2. Correlations between FFR, sRFR, and RFR

**Figure 2:**
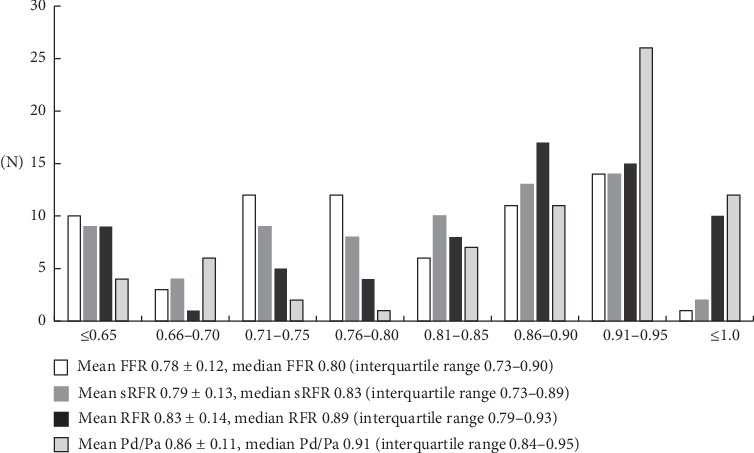
Distributions of pressure measurements in all lesions. RFR, resting full-cycle ratio; FFR, fractional flow reserve; sRFR, saline-induced resting full-cycle ratio.

**Figure 3:**
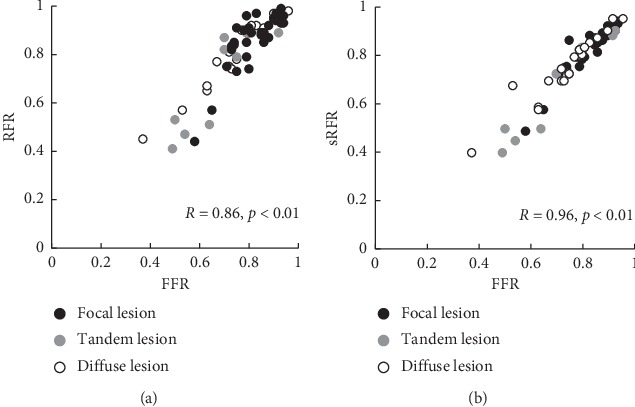
(a) Correlation between the RFR and FFR based on lesion type. (b) Correlation between the sRFR and FFR based on lesion type. Black color (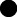), gray color (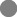), and white color (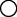) show focal lesion, tandem lesion, and diffuse lesion, respectively. RFR, resting full-cycle ratio; FFR, fractional flow reserve; sRFR, saline-induced resting full-cycle ratio.

**Figure 4:**
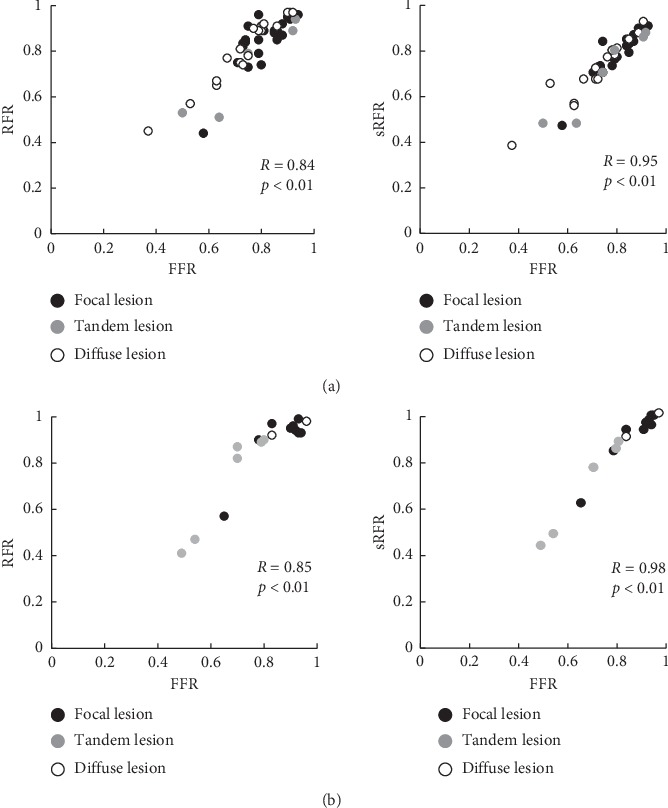
(a) Correlations between the RFR and FFR and sRFR and FFR in the LCA. (b) Correlations between the RFR and FFR and sRFR and FFR in the RCA. Black color (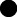), gray color (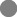), and white color (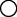) show focal lesion, tandem lesion, and diffuse lesion, respectively. RFR, resting full-cycle ratio; FFR, fractional flow reserve; sRFR, saline-induced resting full-cycle ratio; LCA, left coronary artery; RCA, right coronary artery.

Overall, the mean FFR, mean Pd/Pa, mean sRFR, and mean RFR values were 0.78 ± 0.12, 0.86 ± 0.11, 0.79 ± 0.13, and 0.83 ± 0.14, respectively **(**Figure 2**)**. The median FFR, median Pd/Pa, median sRFR, and median RFR values were 0.80 (interquartile range 0.73–0.90), 0.91 (interquartile range 0.84–0.95), 0.83 (interquartile range 0.73–0.89), and 0.89 (interquartile range 0.79–0.93), respectively. Thirty deferred lesions (43%) were noted. The correlation between the sRFR and FFR was greater than that between the RFR and FFR (*R* = 0.96, *p* < 0.001 versus *R* = 0.86, *p* < 0.01) **(**Figure 3**)**. In addition, there was also a significant correlation between the Pd/Pa and sRFR (*R* = 0.78, *p* < 0.01). These correlations were analyzed for LCA including the LAD and LCX, and RCA. There were also significant correlations between the sRFR and FFR in the LCA and RCA (*R* = 0.95, *p* < 0.01 and *R* = 0.98, *p* < 0.01, respectively) **(**Figure 4**)**.

In addition, significant correlations between sRFR and FFR were observed in not only focal lesions but also in nonfocal lesions including tandem and diffuse lesions (*R* = 0.93, *p* < 0.01 and *R* = 0.97, *p* < 0.01). Furthermore, there were also significant correlations between Pd/Pa and sRFR in not only focal lesions but also in nonfocal lesions including tandem and diffuse lesions (*R* = 0.88, *p* < 0.01 and *R* = 0.76, *p* < 0.01). In addition, significant correlations between Pd/Pa and FFR were observed in not only focal lesions but also in nonfocal lesions including tandem and diffuse lesions (*R* = 0.86, *p* < 0.01 and *R* = 0.73, *p* < 0.01).

Furthermore, there were significant correlations between sRFR and FFR in the nondeferred group and the deferred group (*R* = 0.84, *p* < 0.01 and *R* = 0.54, *p* < 0.01, respectively).

**Figure 5:**
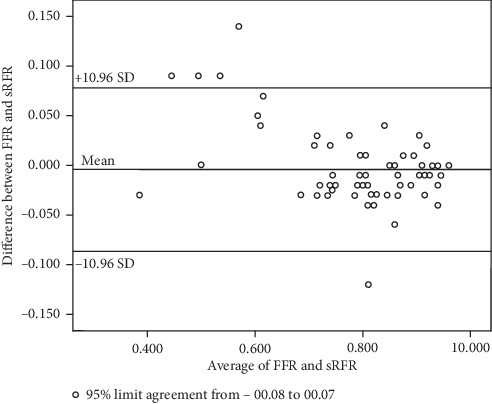
Bland–Altman plot demonstrates close agreement between saline-induced resting full-cycle ratio and fractional flow reserve.

**Figure 6:**
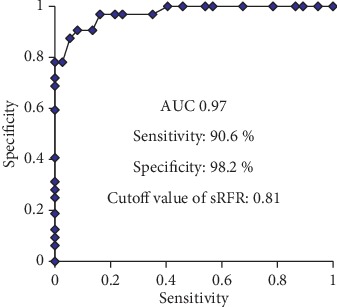
ROC curve analysis for the cutoff value of the sRFR to predict FFR <0.80 (AUC = 0.97; sensitivity: 90.6%; specificity: 98.2%; cutoff value of sRFR: 0.81). ROC, receiver operating characteristic; FFR, fractional flow reserve; sRFR, saline-induced resting full-cycle ratio; AUC, area under the curve.

A close agreement on FFR and sRFR was shown using the Bland–Altman analysis (95% CI of disagreement: −0.08 to 0.07) **(**Figure 5**)**. Furthermore, according to the ROC curve, the sRFR value for predicting a positive FFR value (0.80) was 0.81 (area under curve = 0.97; sensitivity 90.6% and specificity 98.2%) **(**Figure 6**)**. No side effect or complication caused by saline infusion was observed.

## 4. Discussion

The main findings of the present study are as follows: (1) there was a significant correlation between the FFR and sRFR; this was stronger than that between the FFR and RFR and (2) a close agreement between the FFR and sRFR was observed. The ROC curve analysis revealed that the cutoff value of sRFR to predict an FFR of 0.80 was 0.81.

This is the first study to investigate the correlation between the FFR and sRFR.

### 4.1. Effectiveness of the RFR

In the VALIDATE RFR study, the RFR was comparatively analyzed with the instantaneous wave-free ratio (iFR) [13]. As a result, the RFR was diagnostically equivalent to the iFR but unbiased in its ability to detect the lowest Pd/Pa during the full cardiac cycle, potentially unmasking physiologically significant coronary stenosis that would be missed by assessment dedicated to specific segments of the cardiac cycle.

Currently, the iFR is commonly used to assess function of coronary stenosis severity. Two major studies have demonstrated that the iFR is not inferior to the FFR in clinical outcome [14, 15]. Considering these studies, the RFR might also be an effective physiological parameter. In addition, profiles of flow have been reported to differ between in the LCA and RCA [16–18]. These reports suggest that the peak flow in the LCA only occurs during diastole; high intramural pressures are generated in the LCA during systole as the thick left ventricular wall overcomes the perfusion pressure. Although the peak flow in the RCA may also occur very early in diastole, it may rarely occur during systole. Indeed, the RFR in the VALIDATE RFR study was outside the diastole in 12.2% of all cardiac cycles [13]. In addition, the VALIDATE RFR study found that the largest discrepancy occurred when the iFR was >0.93, with the frequency of the discrepancy generally decreasing with lower iFR values. While the discrepancy in the LCA was small, either within or below the iFR “gray zone,” the RFR was detected outside of diastole in 6.5% of cycles in the RCA when the iFR was between 0.86 and 0.93. Furthermore, this discrepancy was only 1.5% when the iFR was ≤0.89 [13]. Thus, although the RFR might be able to detect ischemia that the iFR cannot detect, the benefits of the RFR, especially in the RCA, should be investigated in a future study.

### 4.2. Mechanism of the sRFR

De Bruyne et al. proposed mechanisms of hyperemia induced by continuous intracoronary saline infusion, such as the temperature of saline, decreased local arterial oxygen content, myocardial ischemia, or endothelial paracrine pathways [19]. In addition, a previous study of the saline-induced Pd/Pa ratio has described that the low viscosity effect was also the most probable mechanism of the saline-induced Pd/Pa ratio [9].

There are certain explanations to support the likelihood of this effect. First, De Bruyne et al. reported that the decrease in Pd value began 20  seconds after starting the saline infusion [19]. Meanwhile, in the present study, saline mainly flowed through the epicardial coronary artery during the initial period of three to four heartbeats after starting saline injection and subsequently began to flow through the arterioles. Therefore, decreases in Pd seemed to appear too soon and too rapid to be explained only by hyperemia. Second, a fluid with a lower viscosity can flow through arterioles at a higher rate [20], resulting in its rapid exit from the arterioles to the venous system, with decreased Pd. The blood flow through small vessels is inversely proportional to whole blood viscosity [20], which is affected by volume (haematocrit), deformation and aggregation of red blood cells, and plasma viscosity [21–24]. The viscosities of whole blood at 37°C and saline have been reported to be 4.0–4.5 and 1.012  mPa·s, respectively [20–24]. In their study, Fujimori et al. also found that additional intracoronary boluses of saline immediately after FFR did not affect FFR values, suggesting that the low viscosity effect also does not appear when arterioles are maximally dilated. Although the detailed mechanisms of the sRFR were not examined in the present study, the mechanism of the sRFR may be derived from the theory of lower viscosity and the hyperemia induced by saline infusion, based on previous reports.

### 4.3. Clinical Implication

First, in the present study, no complication caused by saline injection was observed. In the real world, agents to achieve maximal hyperemia for FFR assessment are required, which may sometimes cause complications. A previous study reported that the incidence of complications caused by papaverine, such as ventricular fibrillation, was 1.7% [7]. The present study's finding strongly indicates that the sRFR can be used to easily and accurately predict the FFR safely, irrespective of the lesions type, such as focal or nonfocal (tandem or diffuse). Second, the RFR may be used as an alternative to the resting Pd/Pa ratio, and the iFR may be as a nonhyperemic index to assess the severity of coronary artery stenosis. However, unlike the iFR, the RFR is not limited by sensitive landmarking of components of the pressure waveform or specific to the wave-free period, and thus may have greater clinical utility because of its versatility. Third, although a previous study reported that the iFR pullback predicted the physiological outcome of PCI with a high degree of accuracy [21], there is no report on whether the RFR can predict the same findings as the iFR. Though speculative, if the RFR could be demonstrated to indicate the same findings as the iFR, then the RFR (including the sRFR) may be an important physiological parameter in PCI for culprit lesions such as tandem or diffuse lesions. Last, according to a previous report [22], a contrast-based FFR/FFR hybrid approach showed a significantly low number of lesions requiring adenosine due to a high degree of its accuracy, which might save considerable time and cost. Furthermore, in actual clinical practice, the use of an additional contrast is often not preferred in cases with renal insufficiency. In the present study, the correlation between the FFR and sRFR was significantly stronger than that between the FFR and RFR. In addition, in cases with the RFR “gray zone,” the use of sRFR can not only save considerable time and cost, but also provide a highly accurate method for evaluating ischemia. Considering these findings, sRFR/RFR hybrid approach might be a very useful strategy for evaluating ischemia. Further study will be needed to verify this hypothesis.

### 4.4. Limitations

The limitations of this study are as follows: First, the number of study patients was relatively small. Furthermore, the present study included all lesions such as tandem lesions, which may have been excluded from other registries. However, the accuracy of sRFR remained high, which may be useful in clinical practice. Second, though FFR may not be performed in a case with an RFR value < 0.70 in the real world, the present study included cases showing an RFR value <0.70. Third, in the present case, papaverine was used to measure FFR. The use of papaverine has not been recommended due to the occasional occurrence of ventricular arrhythmia and QT prolongation [23]. However, in CVIT-DEFER registry in Japan, ventricular fibrillation by papaverine was 0.18% at very low [24]. Furthermore, papaverine has also been reported as an alternative agent to induce the maximal hyperemia [23]. Furthermore, according to the Japanese Circulation Society (JCS) 2018 Guideline on revascularization of stable coronary artery disease, papaverine has been recommended as second choice to obtain the maximal hyperemia. Papaverine was therefore selected in the present study. However, adenosine, the gold standard agent for achieving maximal hyperemia, should have been used to avoid some of the abovementioned side effects of papaverine. Although a previous report has suggested that the FFR obtained by adenosine was unstable in a particular case [25], the administration of intracoronary nitroprusside has been reported to be a safe and effective alternative to adenosine [26]. Therefore, an alternative agent to papaverine should have be used in the present study for ensuring safety. Fourth, in the present study, why the correlation between sRFR and FFR in the RCA showed numerically higher correlation compared to those in the LCA remained unclear. In addition, whether the lowest Pd/Pa point was located at the systolic or diastolic phase should have been investigated. Fifth, patients with AF were excluded because the present protocol was based on heartbeats. Sixth, in the present study, saline Pd/Pa or contrast-based FFR values should have been also acquired based on previous reports [8, 9]. In addition, the correlations among the sRFR, saline Pd/Pa, contrast-based FFR, and FFR values should have been compared. In addition, detailed mechanisms of the sRFR should be investigated in the near future. Hence, further study will be needed to overcome these limitations.

## 5. Conclusion

The sRFR can accurately and safely predict the FFR, and it may be effective for diagnosing ischemia, irrespective of lesion types.

## Figures and Tables

**Table 1:** Baseline characteristics and angiographic findings.

	70 lesions
Patients (*n*)	56
Age (years)	72 ± 9
Male/female (*n*)	40/16
BMI	23.1 ± 3.4
Hypertension	44 (78.5)
Smoking, ever	36 (64.2)
Dyslipidemia	39 (69.6)
Diabetes mellitus	28 (50.0)
e-GFR (ml/min)	60.7 ± 17.9
Post PCI	22 (39.2)
Angiographical findings of culprit artery (*n*)	
LAD/CX/RCA	37/15/18
Quantitative coronary angiography	
Minimum lumen diameter (mm)	1.16 ± 0.53
% diameter stenosis (%)	55.0 ± 16.4
Reference diameter (mm)	3.1 ± 0.71
Lesion types of culprit artery	
Focal	40 (57.1)
Diffuse	18 (25.7)
Tandem	12 (17.2)
Deferred lesion	30 (42.8)
Side effect	
VF during FFR	1 (1.4)
VF during sRFR	0 (0)

Data are presented as means ± SD or the number (percentage). BMI, body mass index; e-GFR, estimated-glomerular filtration rate; PCI, percutaneous coronary intervention; LAD, left anterior descending artery; CX, circumflex artery; RCA, right coronary artery; VF, ventricular fibrillation; FFR, fractional flow reserve; sRFR, saline-induced resting full-cycle ratio.

## Data Availability

The datasets generated and/or analyzed during the present study are not publicly available but can be obtained from the corresponding author on reasonable request.
